# In and out among the Hospitals

**Published:** 1888-11-03

**Authors:** 


### In and Out Among the Hospitals.

By a Secretary of Ten Years' Standing.

Copies of Annual Reports and Rules, Accounts of Meetings, Changes in the Staff, &c., should be addressed The Editor, The Lodge, Porchester Square, W.

### HULL ROYAL INFIRMARY.

Most inadequate has been the accommodation afforded for the nursing staff of this infirmary. The want has been pointed out for a long time, and the governors have not been in ignorance of the shortcomings of this institution, so that such a position could only be tolerated as long as it was inevitable. Owing to the energy of the centenary committee, which was appointed to inquire into the matter, the board of management have at last been enabled to risk additional building expenses, and have resolved to convert the Watts wards into a nurses' home the patients who formerly occupied these wards being accommodated in the south-east block, which has just been made available for use. The nurses will therefore now be provided with the comforts due to their position and to the trying nature of their duties. The number of in-patients admitted last year was 1,873, being an increase of 51 on the previous twelve months, while the number of out-patients in the same period was 10,736, an increase of 36. The daily average number of beds occupied was 124. With this good record it is to be regretted that the support hitherto accorded to this charity is declining, but while most sources of income are falling off, it is gratifying to find that the working men's donations continue to increase, and that their efforts during the past year resulted in the largest sum yet realised by them. The income was £8,010 0*s.* 3*d.*, and the expenditure £8,806 1*s.* 1*d.* Deducting 1*s.* each per out-patient, and the extraordinary expenses, the cost per bed occupied was £50 14*s.* 4*d.* A list of the attendances of members of the different committees is given in the report, and shows at a glance to whom the governors are indebted for the management of the infirmary.

### THE FRENCH HOSPITAL AND DISPENSARY.

It is generally supposed that this hospital is for the exclusive treatment of French subjects, but this idea is erroneous, as all foreigners are treated within its walls. There is perhaps no other charity, except the Dreadnought Seamen's Hospital, that is of such a cosmopolitan nature. The greater number of the patients are indeed French, but there are also a large number of Italians, Swiss, Belgians, and Germans. The in-patients numbered 2,422, and the out-patients 3,315. The expenditure was £2,541 16*s.* 7*d.*, and the receipts exceeded this by £1,009 18*s.* 10*d.*, which has enabled the management to invest the sum of £819. Deducting 1*s.* per out-patient, the cost per bed occupied was £84 4*s.* 5*d.* The dietary of the patients is a liberal one, and contains two items to which we are unused, viz., vegetable soup for breakfast at eight o'clock, and half a-pint of wine for dinner. The patients are nursed by sisters of charity.

